# Quantitative Assessment of Intra-Patient Variation in CD4+ T Cell Counts in Stable, Virologically-Suppressed, HIV-Infected Subjects

**DOI:** 10.1371/journal.pone.0125248

**Published:** 2015-06-25

**Authors:** Claire L. Gordon, Allen C. Cheng, Paul U. Cameron, Michael Bailey, Suzanne M. Crowe, John Mills

**Affiliations:** 1 Department of Infectious Diseases, The Alfred hospital, Melbourne, Australia; 2 Department of Epidemiology and Preventative Medicine, Monash University, Melbourne, Australia; 3 Macfarlane Burnet Institute for Medical Research and Public Health, Melbourne, Australia; 4 Department of Medicine, Monash University Central Clinical School, Melbourne, Australia; University of Toronto, CANADA

## Abstract

**Objectives:**

Counts of absolute CD4+ T lymphocytes (CD4+ T cells) are known to be highly variable in untreated HIV-infected individuals, but there are no data in virologically-suppressed individuals. We investigated CD4+ T cell variability in stable, virologically-suppressed, HIV-1 infected adults on combination antiretroviral therapy (cART).

**Methods:**

From a large hospital database we selected patients with stable virological suppression on cART for >3 years with >10 CD4+ T cell measurements performed over a further >2 years; and a control group of 95 patients not on cART.

**Results:**

We identified 161 HIV-infected patients on cART without active HCV or HBV infection, with stable virological suppression for a median of 6.4 years. Over the study period 88 patients had reached a plateau in their absolute CD4+ T cell counts, while 65 patients had increasing and 8 patients had decreasing absolute CD4+ T cell counts. In patients with plateaued CD4+ T cell counts, variability in absolute CD4+ T cell counts was greater than in percent CD4+ T cells (median coefficient of variation (CV) 16.6% [IQR 13.8-20.1%] and CV 9.6% [IQR 7.4-13.0%], respectively). Patients with increasing CD4+ T cell counts had greater variability in absolute CD4+ T cell counts than those with plateaued CD4 T cell counts (CV 19.5% [IQR 16.1-23.8%], p<0.001) while there was no difference in percent CD4+ T cell variability between the two groups. As previously reported, untreated patients had CVs significantly higher than patients on cART (CVs of 21.1% [IQR 17.2-32.0%], p<0.001 and 15.2% (IQR 10.7-20.0%), p<0.001, respectively). Age or sex did not affect the degree of CD4+ variation.

**Conclusions:**

Adults with stable, virologically-suppressed HIV infection continue to have significant variations in individual absolute CD4+ T cell and percent CD4+ T cell counts; this variation can be of clinical relevance especially around CD4+ thresholds. However, the variation seen in individuals on cART is substantially less than in untreated subjects.

## Introduction

The decision to commence combination antiretroviral therapy (cART) for patients with asymptomatic human immunodeficiency virus-1 (HIV) infection may be based primarily on the absolute CD3+CD4+ T lymphocyte count (CD4+ T cell count), although the percent CD4+ T cells and HIV viral load may also be considered [1]. Following initiation of cART, the CD4+ T cell count continues to be monitored because decisions regarding commencement or continuation of prophylaxis against a range of opportunistic infections (OI) are based on the level of immune reconstitution that is achieved; in some resource-limited environments CD4+ testing may be continued to monitor response to cART [2, 3].

Several studies have reported wide, intra-individual variability in CD4+ T cell counts in treatment-naïve, HIV-infected individuals due to both laboratory and physiological factors [4–6]. This variability, up to 18–25%, limits the utility of a single CD4+ T cell measurement for clinical decision-making [4, 7, 8]. This issue is particularly problematic in resource poor settings where often only a single CD4+ T cell count is available prior to initiating therapy. Absolute CD4+ T cell counts are affected by variables such as age, season, ethnic origin, the time of day the sample was taken, exercise, smoking and inter-current infection [8–13]. However, it is rarely possible to control for these variables in a busy outpatient setting. In addition, the laboratory process of CD4+ T cell testing has inherent imprecision, particularly related to pipetting errors, during quantification of both total lymphocyte count and to a lesser degree the percent CD4+ T cell number [6, 8].

Long-term CD4+ variability in stable, virologically-suppressed, HIV-infected patients on cART has not been reported. We have documented the variation in individual patient’s absolute and percent CD4+ T cell values in HIV-infected subjects without active HBV or HCV infection (“HIV mono-infected) in the setting of sustained, long-term virologic suppression, and have compared these subjects with individuals with untreated HIV infection (solely for reference with previous data on these latter subjects). The CD4+ T cell assays were conducted by a single accredited flow cytometry facility in an academic teaching hospital. A nomogram provides a clinical guideline relevant to this population as to what constitutes significant changes outside of the observed range of variability in absolute and percent CD4+ T cell number over time.

## Materials and Methods

This study was approved by the Human Research and Ethics Committee (HREC) of The Alfred Hospital/Bayside Health. The Alfred HREC explicitly allowed this study to be performed without obtaining consent from individual patients. Consent was not obtained from individual patient because the data were all anonymized, de-identified prior to analysis, and aggregated prior to publication. The database was anonymized by Ms. Kerrie Watson, the Manager of the Alfred Hospital HIV Database (see Acknowledgments in the manuscript), and she also protected the database with a password. Further, none of the authors have interacted, in any way, with the subjects (patients) in the study.

### Selection of subjects

Retrospective data including clinical characteristics and laboratory results were extracted from a large research database (4551 subjects) of all HIV-infected patients seen by The Alfred Hospital’s HIV Clinical Service in Melbourne, Australia, from January 2005 to May 2010, and were corroborated by direct reference to clinical and laboratory information in the patient’s hospital record. Patients were included in the study if they fulfilled the following inclusion criteria: (1) on continuous effective cART (not necessarily the same regimen) for ≥3 years and long-term (≥3 years) continuous suppression of HIV replication as defined by HIV viral load values consistently ≤50 copies/ml but allowing for ≤3 non-consecutive results of 50–500 RNA copies/ml (“blips”); and (2) those who after 3 years of virologic suppression had ≥10 CD4+ T-cell measurements performed over an additional period of ≥2 years. For this study CD4+ T cell measurements were included in the analysis only after 3 years of virologic suppression on cART had been achieved. We focused our study on CD4+ measurement obtained from patients with sustained virologic suppression after at least 3 years of cART, because prior investigators have shown that the rate of CD4+ change is substantially slower beyond 3 years than during the first 3 years of therapy [14]. Patients were further divided into three groups defined by statistically-different trends in their CD4+ T cell measurements over the study period (i.e. those with a plateau (slope = zero), declining (slope <0), or inclining (slope>0). The main analysis was performed in patients who had reached a plateau in their CD4+ T cell counts. Patients with active HBV and/or HCV infection were excluded because of the potential effect of hepatitis virus co-infection on CD4+ T cell variability [15]. CD4+ T cell counts and HIV viral load tests were obtained at approximately 3-monthly intervals, in accordance with national management guidelines [16]. To directly compare our data regarding patients on cART with prior studies of untreated HIV-infected patients we also identified a cohort of untreated patients from the same research data base over the same time period who had ≥10 CD4+ T-cell measurements over ≥2 years.

### Flow cytometry

Flow cytometric analyses were performed using a single platform assay (Beckman Coulter TetraONE) at The Alfred hospital, a laboratory certified by Australian National Association of Testing Authorities (NATA) and participating in the Royal College of Pathology of Australasia (RCPA) quality-assurance program. In the laboratory the precision of the assay for CD4+ T cell count (CD3+CD4+ T cells gated for lymphocytes) and percent CD4+ T cells were 5.5% and 1.4%, respectively; compared to other laboratories in the RCPA program the values were 8.5% and 4.8%. Assessment of HIV viral load was performed using the Roche COBAS Amplicor Monitor 1.5 ultrasensitive method in the Burnet Institute’s Clinical Research Laboratory, which is on the same campus as The Alfred hospital and is also NATA and ISO15189 accredited.

### Statistical analysis

A linear regression was performed for each patient’s CD4+ cell count over time, and the error between observed and model predicted CD4+ cell count (both absolute and percentage) was used to calculate a standard deviation for each patient. As the standard deviation was found to correlate with each patient’s mean absolute or percent CD4+ T cell count, results were expressed as a coefficient of variation (CV). The relationship between CV of absolute or percent CD4+ T cell count and mean absolute or percent CD4+ T cell count was compared using linear regression on log transformed CV to ensure normal distribution. Continuous measures were compared using Student’s t-test or Wilcoxon rank-sum test for normally-distributed and non-parametric data respectively. The change in CD4+ T cells over time for each patient was assessed using linear regression with the statistical significance of the decline in slope determined using Student’s t-test. Patients with significantly negative or positive slopes were compared against patients with plateaued slopes. Relationships between non-normally distributed continuous variables were determined using Spearman correlation coefficients. Analysis was performed using STATA version 11.0 (StataCorp. College Station, TX: StataCorp LP) and SAS version 9.2 (SAS Institute Inc., Cary, NC, USA) A p value of ≤0.05 was considered significant.

## Results

We identified 203 subjects (187 males, 16 females) fulfilling the inclusion criteria of ≥3 years of cART, continuous, long-term HIV virological suppression for ≥3 years, followed by ≥10 CD4+ T cell measurements. Thirteen patients with HBV-co-infection and 29 patients with HCV-co-infection were subsequently excluded, leaving only HIV mono-infected subjects (n = 161). Ninety-five subjects not on cART and with similar number and duration of CD4+ assays were also identified and examined as controls.

**Fig 1:**
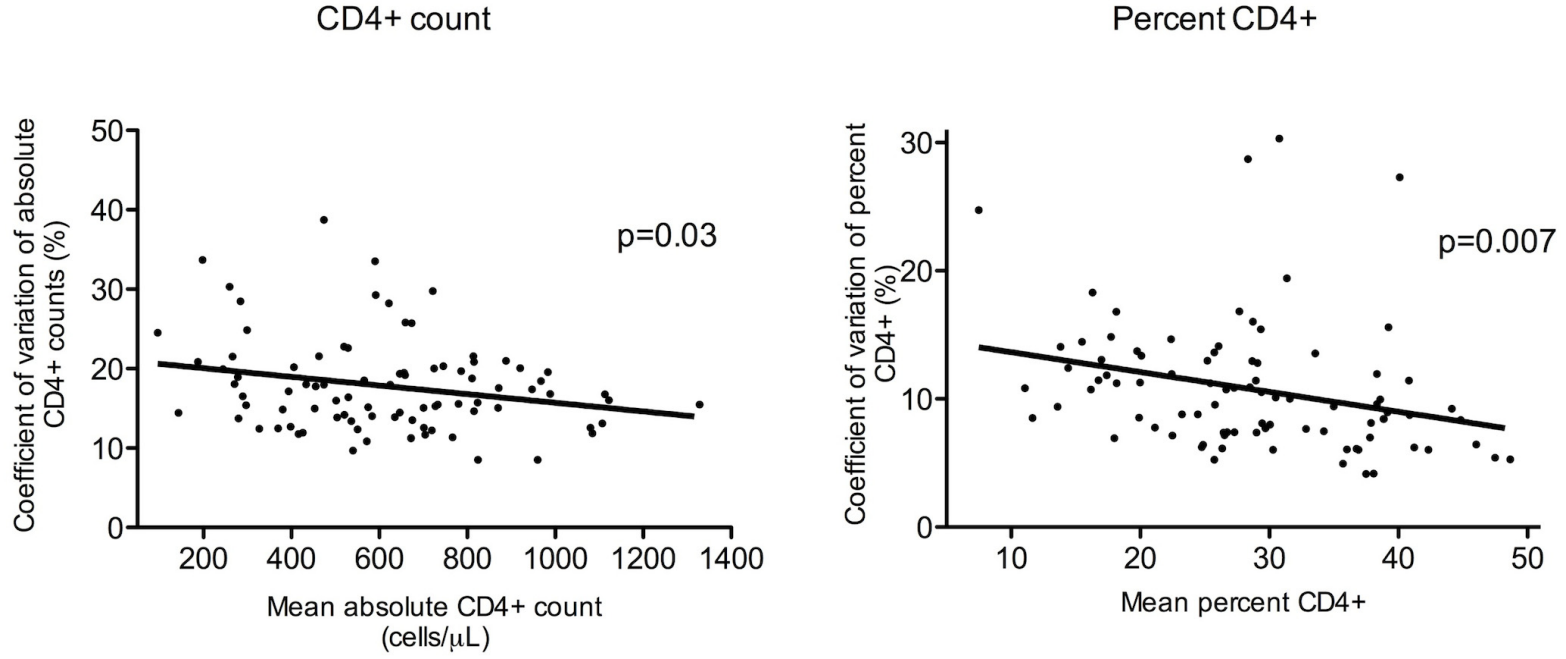
Coefficient of variation of absolute and percent CD4+ T cell counts in relation to the mean for the individual HIV-infected subjects. The CV of individual absolute and percent CD4+ T cell counts were derived from a median of 17 CD4+ T cell counts obtained over a median of 4.8 years, in a cohort of 88 adult patients with HIV mono-infection who had been on cART with suppressed viral loads and plateaued CD4+ T cell counts for a median of 6.4 years.

The 88 adult HIV-infected patients on cART and plateaued CD4+ T cell counts were predominantly male (reflecting the Australian HIV+ population) and had been on cART for a median of 6.4 years (range 3 to 12 years) with suppressed viral loads for at least the previous 3 years at the time of the first CD4+ measurement used for the analysis (Table 1). These subjects contributed a median of 17 CD4+ T cell counts over a median of 4.8 years to the analysis (Table 1). There was wide variation in both absolute and percent CD4+ T cell counts (Table 1). Even greater variability in absolute CD4+ T cell counts occurred in the group with increasing CD4+ T cell counts (n = 65) compared to the group with plateaued CD4+ T cell counts (CV 19.5% [IQR 16.1–23.8%] vs. CV 16.6% [IQR 13.8–20.1%]), p<0.001). This difference in variability was not seen with percent CD4+ T cells (CV 10.3% [IQR 7.7–13.4% vs. CV 9.6% [IQR 7.4–13.0%]], p = 0.5). The CV for absolute CD4+ T cell number or CD4+ percentage tended to decrease slightly as the mean absolute CD4+ count or mean percent CD4+ increased (Fig 1).

**Table 1:** Demographics, clinical details and CD4+ count variability of patients with suppressed HIV infection on ART and untreated HIV infection.^§^

	Treated HIV infection† (n = 88)	Untreated HIV infection (n = 95)
*Demographics*		
Median age in years (IQR)	50 (42–57)	33 (27–40)*
Male sex number (%)	80 (91%)	86 (91%)
Median years on cART (IQR)†	6.4 (3.5–8.2)	-
Median no. of CD4+ T cell counts (IQR)	17 (14–20)	14 (11–17) *
Median years CD4+ T cell counts collected (IQR)	4.8 (3.6–5.0)	5.3 (3.4–8.2) *
*CD4+ T cell counts*		
Mean of individual’s absolute CD4+ T cell count ± SD	616 ± 265	593 ± 265
Median CV of CD4+ T cell count (IQR)	16.6 (13.8–20.1)	21.1 (17.2–32.0) *
*Percent CD4+*		
Mean of individuals’ percent CD4+T cell count	28.6 ± 9.2	27.6 ± 8.2
Median CV of percent CD4+ T cell count (IQR)	9.6 (7.4–13.0)	15.2 (10.7–20.0)*

^†^Patients with active HCV or HBV infection were excluded. Patients were on ART for a minimum of 3 years and had reached a plateau in their CD4+ T cell counts.

^§^Legend: Coefficient of variation (CV); standard deviation (SD); interquartile range (IQR);

* p <0.01 when compared to treated HIV infection; Duration of cART was calculated from the date cART started to the date of the first CD4+ measurement used in the analysis.

**Fig 2:**
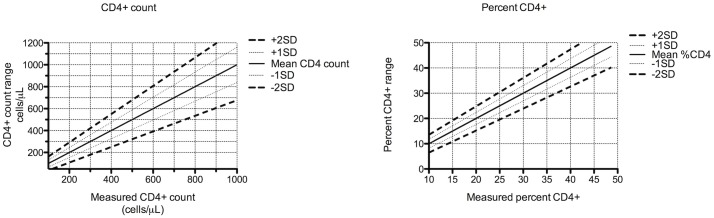
Estimated range of absolute and percent CD4+ T cell counts with a single determination The predicted confidence limits of absolute and percent CD4+ T cell count based on a single recorded CD4+ T cell measurement from 88 HIV-infected individuals (same cohort as in Fig 1). Sixty-eight % of mean absolute or percent CD4+ T cell counts will fall within ± 1 standard deviation (SD) from the mean; 95% of mean absolute or percent CD4+ T cell counts will fall within ± 2 SD from the mean.

There was significant inter-patient variation in CD4+ measurements, with the lowest CV for absolute and percent CD4+ T cell counts being 8.5% and 4.2% respectively, while the highest CV was 39.7% and 30.3%, respectively. To further explore the clinical heterogeneity observed in patients with a plateaued CD4+ T cell count, we investigated the relationship between CD4+ T cell count variability and factors which may affect it, including age, sex, duration of cART (after an initial 3 years of therapy), slope of CD4+ T cell counts over time, low initial CD4+ count (defined by the clinically relevant cut offs of ≤350 cells/mm3 for low initial CD4+ T cell count and >500 cells/mm3 for high initial CD4+ T cell count [17]), and the existence of outlier CD4+ count values acting as “leverage points” (very high or very low, outlier CD4+ T cell counts) on either end of the study period. There was no relationship between sex or age and CD4+ T cell variation (data not shown). Further, there was no correlation between duration of cART (after an initial 3 years of therapy) and absolute or percent CD4+ T cell count variability (Spearman rho = 0.12, p = 0.28; Spearman rho = 0.07, p = 0.53, respectively). Subjects with a low initial absolute CD4+ T cell count (≤350 cells/mm3, n = 14) at the start of the study period had greater CD4+ T cell variability compared to subjects with a high initial absolute CD4+ T cell count (≥500 cells/mm3, n = 54; median CV 22.2% [IQR 18.1–28.2%] vs. CV 16.0% [IQR 13.8–18.1%], p = 0.02, respectively). We reviewed five patients with the steepest decline and five patients with the steepest incline in CD4+ slopes and did not find leverage points at either end. The predicted confidence limits of absolute and percent CD4+ T cell count based on a single recorded CD4+ T cell measurement from a patient with stable, virologically suppressed HIV infection are summarized in Fig 2. Patients with plateaued absolute CD4+ T cell counts and a mean absolute CD4+ count around the thresholds of 350 (±75 cells/mm3, n = 13) and 500 (±50 cells/mm3, n = 13) had similar CD4+ count variability (15.4% [IQR 12.7–18.2} vs. 16.4% {IQR 14.2–21.6], p = 0.5, respectively).

The 95 control subjects with untreated HIV infection were younger and had fewer CD4+ measurements taken, but these were collected over a longer time period than the treated subjects. These patients had a similar mean absolute CD4+ count and percent CD4+ to treated patients (Table 1). The mean HIV log10 VL for the naïve cohort was 3.81 (±0.8 SD) copies /ml. The CD4+ variation in this group was significantly higher than that observed in the treated patients (Table 1). Similar to the cART treated group, in the naïve patients variability decreased as mean absolute CD4+ T cell number and mean percent CD4+ increased (p<0.001 and p<0.001, respectively)

To determine whether there were intra-individual changes in the CD4+ T cell CV over the study period, we analysed data from all subjects with ≥16 CD4+ measurements (n = 83, median CD4+ count 20 [IQR 18–21]) and compared the CV in the first half of the study period to that in the second half. The CV of absolute CD4+ T cell number (15.8% vs 16.1%, p = 0.78) and percent CD4+ T cells (10.3% vs. 10.8%, p = 0.52) did not vary between the two study periods confirming that the CD4+ T cell variability in individual patients was stable over time.

## Discussion

Our study shows that even in adults on cART with long-term, sustained virologic suppression and plateaued CD4+ T cell counts there is still wide variation in individual absolute CD4+ T cell counts over time (CV of 16.6% [IQR 13.8–20.1%]) with less, but still significant, variation in percent CD4+ (CV 9.6% [IQR 7.4–13.0%]). To our knowledge this is the first study to describe the variability in individual patient CD4+ T cell counts in HIV-infected adults on cART with long-term virologic suppression. These data would suggest that decisions based on a single CD4+ T cell count–to treat HIV infection or to discontinue OI prophylaxis–should consider using a higher absolute CD4+ number (e.g. 233) instead of 200 CD4+ T cells/μl when considering discontinuing OI prophylaxis) to ensure that all subjects with the threshold CD4+ count are included (see Fig 2). In settings where HIV viral load testing is readily available, our data describing significant variability in CD4+ T cell measurements supports a recent move away from CD4+ T cell monitoring towards HIV VL monitoring of response to cART [18, 19]. Although a single CD4+ measurement has been used with clinical success in the past to define CD4+ count thresholds for both starting and discontinuing OI prophylaxis [20], we know more about flow cytometric imperfections and biological variability and further definition of CD4+ counts with new data including the findings of this study may further improve outcomes.

We found that CD4+ variability was substantially less among patients on cART than in a treatment-naïve cohort from the same hospital analysed simultaneously (CVs of 21.1% [IQR 17.2–32.0] and 17.6% [IQR 14.5–21.6] for absolute counts and percent, respectively, in our naïve cohort). Our data on CD4+ T cell count variation in untreated patients is entirely consistent with previous studies of treatment-naïve patients with reported CVs of 19–25% for absolute [4, 7, 12] and 11–18% for percent CD4+ T cell counts [7, 12].

Our study cohort of patients with prolonged virologic suppression was intentionally clinically heterogeneous to reflect the patient population seen by physicians who may have access to one or a few CD4+ T cell measurements. Additional exploration of the clinical heterogeneity of the study group showed that greater CD4+ T cell count variability occurred in patients with low initial CD4+ T cell count or that had increasing CD4+ T cell counts over the study period, consistent with the expected variability in CD4+ count measurements as CD4+ T cells recover. Nevertheless, in patients with plateaued CD4+ T cell counts, CD4+ T cell variability was still high (CV 16.6%).

CD4+ T cell variability on serial testing is the sum of the error associated with the assay plus biological alterations in the patient. In contrast to earlier studies that showed a high variation in laboratory assays, current flow cytometric assays, particularly single platform assays as used for this study, provide more reproducible CD4+ T cell results; and further, most clinical laboratories now participate in quality assurance (QA) programs [6, 21, 22]. The Alfred Hospital flow cytometry facility had intra-laboratory variation of 5.5% and inter-laboratory variation of 8.5% for CD4+ T cell counts in their Australian-based QA program. Given our mean variation on CD4+ counts for treated, stable, HIV mono-infected subjects was 16.6%, the estimated contribution of biological factors to CD4+ T cell count variability in this population is in the range of 8.1–11.1% (16.6% minus the variation due to flow cytometric analysis)—or about 60% of the total variation. Approaches to reducing CD4+ T cell count variability include drawing blood at a similar time of day, meticulous laboratory technique, using single platform rather than double platform technology and using serial test results to determine trend [23]. However, even in an academic hospital, busy clinics and clinical laboratories will find it virtually impossible to control for all of these variables; and furthermore, the direction of the effect for each variable is unpredictable. Percent CD4+ T cell count has substantially less variability than absolute CD4+ number and although essential for monitoring pediatric infection, it is not widely available in developing countries. Although CD4+ T cell counts are known to have significant diurnal variation, especially between 08:00 and 22:00 [12], this effect was likely to have been minimal in this study as no difference in timing of blood collection (nearly all blood samples in the study were taken between 08:00 and 12:00) taking was observed in participants who had high CD4+ T cell count variability and those with low variability.

To aid the HIV clinician, a nomogram (Fig 2) predicts the range of mean CD4+ T cell counts in this population for a single absolute or percent CD4+ T cell result allowing the clinician to estimate the 95% confidence limits (± 2 SD) of a single CD4+ T cell count in an individual patient. This diagram could be especially useful in determining whether a patient’s true CD4+ T cell count may have actually fallen below a critical number (e.g. 200, 350 or 500 cells/μl).

Our study is limited by the fact that we analysed a relatively small cohort of patients, all adults, largely male, mainly Caucasian and predominantly men who have sex with men, from a single hospital-based clinic in Australia. Consequently, the results from this cohort may not be generalizable to other patient groups. The untreated patients may be enriched for relatively slow progressors as those with rapid progression may not have had the requisite 10 observations prior to cART initiation. This feature may have resulted in the CD4+ T cell count variability in the treatment naïve group being underestimated; however, we observed no difference in CD4+ variability between subjects with only 10 CD4+ measurements and those with more than 19 CD4+ measurements to support this hypothesis (data not shown). The rate of change in CD4+ count on cART depends on a variety of factors including cART duration, CD4+ count at cART initiation, and current CD4+ count. CD4+ T cell counts can continue to increase even up to five years after cART initiation [14]. Hence, the selection of a 3-year time of cART cut-off may not take into consideration relevant covariates.

CD4+ counts may be useful for starting cART in developed countries, and are almost universally required in developing countries where patients must have counts below a government- or WHO-specified threshold in order to receive cART. Further, in the many areas without access to viral load testing, CD4+ counts are often used to assess the success of cART, and the decision to commence or change opportunistic infection (OI) prophylaxis invariably requires CD4+ counts. In resource limited countries decisions about starting cART (and where used, OI prophylaxis) are often based on a single CD4+ T cell count, the variability of which is nearly impossible to control in real life. Of note, for some OIs, OI prophylaxis may be safely stopped at lower CD4+ T cell counts provided the VL is fully supressed on therapy [24]. Our nomogram of estimated absolute and percent CD4+ T cell count for a single CD4+ T cell measurement may assist clinicians in using CD4+ T cell data when managing patients.
